# Obituary of Dr. W. R. Hodgen

**Published:** 1866-01

**Authors:** A. S. Talbert, G. W. Priest


At a called meeting of the dental profession in the city of Lexington, Ky., to take into consideration such action as may be appropriate to the memory of our deceased brother and friend, Dr. W. R. Hodgen, Dr. A. S. Talbert was called to the Chair, and Dr. G. W. Priest requested to act as Secretary. The Chairman appointed Drs. R. J. Poore, J. Holmes, and J. J. Wilson a committee to draft such resolutions as would be appropriate to the occasion. The following preamble and resolutions were unanimously adopted:

Whereas, It has pleased an All-wise Providence to take from our midst, in the beginning of manhood, of social and professional usefulness, Dr. W. R. Hodgen—therefore,

*Resolved*, That we, the members of the Dental profession of this city, take pleasure in giving our testimony to the moral worth, genial disposition, and upright character of the deceased.

*Resolved*, That we tender the stricken family and friends our heartfelt condolence and sympathies, assuring them that through the evidence of his life, what has proven their loss, is his eternal gain.

*Resolved*, That the papers of our city and the Dental Register of Cincinnati, be requested to publish these resolutions, and that a copy of the same be transmitted to the bereaved family, by the Secretary.

A. S. TALBERT, President.

G. W. Priest, Secretary.

